# PlanAct: An eclipse scripting API‐based module embedding clinical optimization strategies for automated planning in locally advanced non‐small cell lung cancer

**DOI:** 10.1002/acm2.70304

**Published:** 2025-10-09

**Authors:** Hao Guo, Tenzin Kunkyab, Yang Lei, Kenneth Rosenzweig, Robert Samstein, Ming Chao, Tian Liu, Junyi Xia, Jiahan Zhang

**Affiliations:** ^1^ Department of Radiation Oncology Icahn School of Medicine at Mount Sinai New York USA

**Keywords:** knowledge‐based planning

## Abstract

**Background:**

Manual intensity‐modulated radiotherapy (IMRT) planning for locally advanced non‐small cell lung cancer (LA‐NSCLC) is labor‐intensive and time‐consuming. Knowledge‐based planning (e.g., RapidPlan) improves consistency but commonly falls short in fully meeting clinical objectives in LA‐NSCLC cases, requiring iterative manual adjustments.

**Purpose:**

To develop and validate PlanAct, an Eclipse Scripting API (ESAPI)‐based module for optimizing automated IMRT planning in LA‐NSCLC, and to compare its performance against clinical and RapidPlan‐generated plans across a retrospective patient cohort.

**Methods:**

PlanAct was developed with modular functions to automate key tasks in IMRT plan generation and optimization. PlanAct was manually executed on 56 anonymized retrospective LA‐NSCLC cases using a standardized nine‐beam geometry. Plans were normalized to ensure 95% planning target volume (PTV) coverage. The PlanAct‐optimized plans were evaluated against RapidPlan‐generated plans and clinically approved plans using institutional plan quality metrics, including dose‐volume constraints for the esophagus, spinal cord, lungs, heart, larynx, and PTV. Statistical comparisons were performed to assess differences in plan quality and unmet dosimetric requirements.

**Results:**

PlanAct‐optimized plans demonstrated significant improvement in plan quality compared to RapidPlan, with fewer unmet clinical requirements and better organ‐at‐risk sparing, particularly for the lungs (*p* < 0.001 for V_20_ and D_mean_). Only one PlanAct‐optimized plan failed to meet all dose constraints (in this case, lungs D_mean_) due to a large PTV volume, compared to 18 RapidPlan and 10 clinical plans. Even in anatomically challenging cases, PlanAct produced more favorable dose distributions, with superior hotspot control.

**Conclusions:**

PlanAct is an effective tool to optimize automated IMRT planning in LA‐NSCLC. It produced plans comparable to or better than clinical plans, even in challenging cases. Its modular architecture makes it promising for integration into future fully autonomous, patient‐specific radiotherapy treatment planning systems.

## 1 INTRODUCTION

Radiotherapy has been the primary treatment or a critical treatment component for locally advanced non‐small cell lung cancer (LA‐NSCLC), with intensity‐modulated radiotherapy (IMRT) and volumetric‐modulated arc therapy (VMAT) increasingly utilized for their ability to enhance dose conformity, spare normal tissues, and minimize radiation exposure to organs at risk (OARs).^1^, ^2^, ^3^ Manual IMRT planning for lung cancer is widely recognized as labor‐intensive and time‐consuming. It relies on trial‐and‐error adjustments by experienced planners and meticulous coordination among multidisciplinary professionals, in extreme circumstances requiring multiple days of back‐and‐forth iterations.^4^ As a result, achieving consistent high‐quality IMRT plans is challenging due to its complexity and demand of high expertise.^5^, ^6^

Automatic treatment planning is a promising strategy to enhance efficiency, consistency, and quality for IMRT by reducing manual workload. Started in 2003 and grew rapidly after 2011 (when commercial softwares became available), automation in IMRT treatment planning continues to attract researchers without a sign of saturation.^7^ A representative commercial software is Varian's RapidPlan. Leveraging prior planning knowledge embedded in existing treatment plans, RapidPlan generates (believed) best achievable DVH estimations. These estimations are subsequently used for placing optimization constraints for new patients, ensuring consistent and high‐quality plans. Studies show RapidPlan improves organ‐at‐risk sparing and target coverage compared to manual planning.^8^, ^9^ However, it is common that RapidPlan falls short in generating a plan which fully achieves clinical planning objectives, and planners usually need to take extra manual optimization steps, forgoing some of the time‐saving benefits. This is especially the case for lung IMRT planning, where dose calculation differences between the dose engine used by the optimizer (e.g., Varian's Fourier Transform Dose Calculation algorithm) and the final dose calculation algorithm (AAA or Acuros) often lead to multiple iterations of plan modifications after running RapidPlan.

Automation through scripting frameworks like the Eclipse Scripting Application Programming Interface (ESAPI) represents another path toward improved IMRT planning efficiency.^10^ ESAPI converts user interactions in the treatment planning system into executable function calls and enables end‐users to automate key tasks commonly taken by the planners. It has been shown that ESAPI can be effectively combined with RapidPlan and multi‐criteria optimization (MCO).^11^

We hypothesize that integrating ESAPI with RapidPlan can unlock new efficiencies and improve workflow adaptability in lung IMRT planning by automating repetitive tasks and incorporating institution‐specific expertise. In this study, we aim to replicate expert strategies with ESAPI script to standardize optimization and further improve plan quality and planning efficiency. We developed and validated an ESAPI‐based module named PlanAct, designed to automate critical tasks in further optimizing IMRT plans generated by knowledge‐based planning (KBP). We retrospectively evaluated the effectiveness of PlanAct with lung cases, systematically comparing its dosimetric performance against both clinical and RapidPlan‐generated IMRT plans. Our goal was to demonstrate PlanAct's capability to consistently meet or exceed established clinical dosimetric objectives, reduce unmet planning requirements, and enhance the sparing of critical structures, particularly in anatomically complex cases.

## 2 METHODS AND MATERIALS

### 2.1 ESAPI‐based script module

PlanAct consists of modular ESAPI‐based scripts systematically categorized into four functional groups (Table 1). Group 0 initializes the treatment plan using a clinical RapidPlan model fine‐tuned at our institute, generating optimization objectives tailored to the patient.^12^ Group 1 focuses on improving dose coverage and reducing mean dose to critical organs by refining dose distributions. Group 2 addresses the reduction of maximum doses by applying targeted constraints to mitigate hotspots. Group 3 finalizes the plan using Eclipse‐based optimization and normalization to ensure clinical goal compliance.

**TABLE 1:** Modular ESAPI‐based scripts contained in PlanAct.

Group	Script name	Description	Input parameters
0	Initializer	Generates an initial treatment plan per prescription.	Plan type, OAR preference, preference level
	NormalizeDmean	Normalizes the plan based on the mean dose within the PTV.	PTV structure, D_mean_ objective
1	ReduceROIMean	Reduces the mean dose to a specific region.	OAR structure, target dose level, constraint weighting
	IncreaseCoverage	Increases dose coverage on underdosed areas.	PTV structure, isodose level, target dose level, constraint weighting,
2	ReduceBodyDmax	Reduces the maximum dose outside the target.	
	ReducePTVDmax	Reduces the maximum dose within the PTV.	
	ReduceDvolume	Reduces dose delivered to a specific structure volume.	PTV structure, OAR structure, dose level, target dose level, constraint weighting
3	OptimizePlan	Implements the Eclipse optimization algorithm.	Plan type, optimization mode
	NormalizePlan	Normalizes the treatment plan to desired dose level.	PTV structure, PTV coverage

Each script (action) within the PlanAct module aligns with clinical objectives by replicating common human planner strategies. The Initializer script utilizes a clinical RapidPlan model to define optimization constraints for the current patient and optimize the plan, creating an initial plan aligned with clinical objectives and previous planning knowledge. The ReduceROIMean script introduces a mean‐dose upper constraint to a structure. IncreaseCoverage functions by defining an isodose‐derived substructure within the planning target volume (PTV) based on prescription percentages, identifying underdosed regions, and applying lower‐dose constraints to enhance uniformity and coverage. Scripts ReduceBodyDmax and ReducePTVDmax create substructures within the body or PTV using dose thresholds and subsequently apply upper dose constraints to mitigate hotspots. Lastly, the ReduceDvolume script ensures adherence to clinical dose‐volume constraints by applying precise upper‐dose limitations to target structures, thereby maintaining compliance with established clinical dosimetric standards. Each action is followed by a two‐stage optimization, which approximately took 3 min for IMRT plans. Pseudo‐code blocks of each PlanAct action are presented in the supporting materials.

### 2.2 Study workflow

**FIGURE 1:**
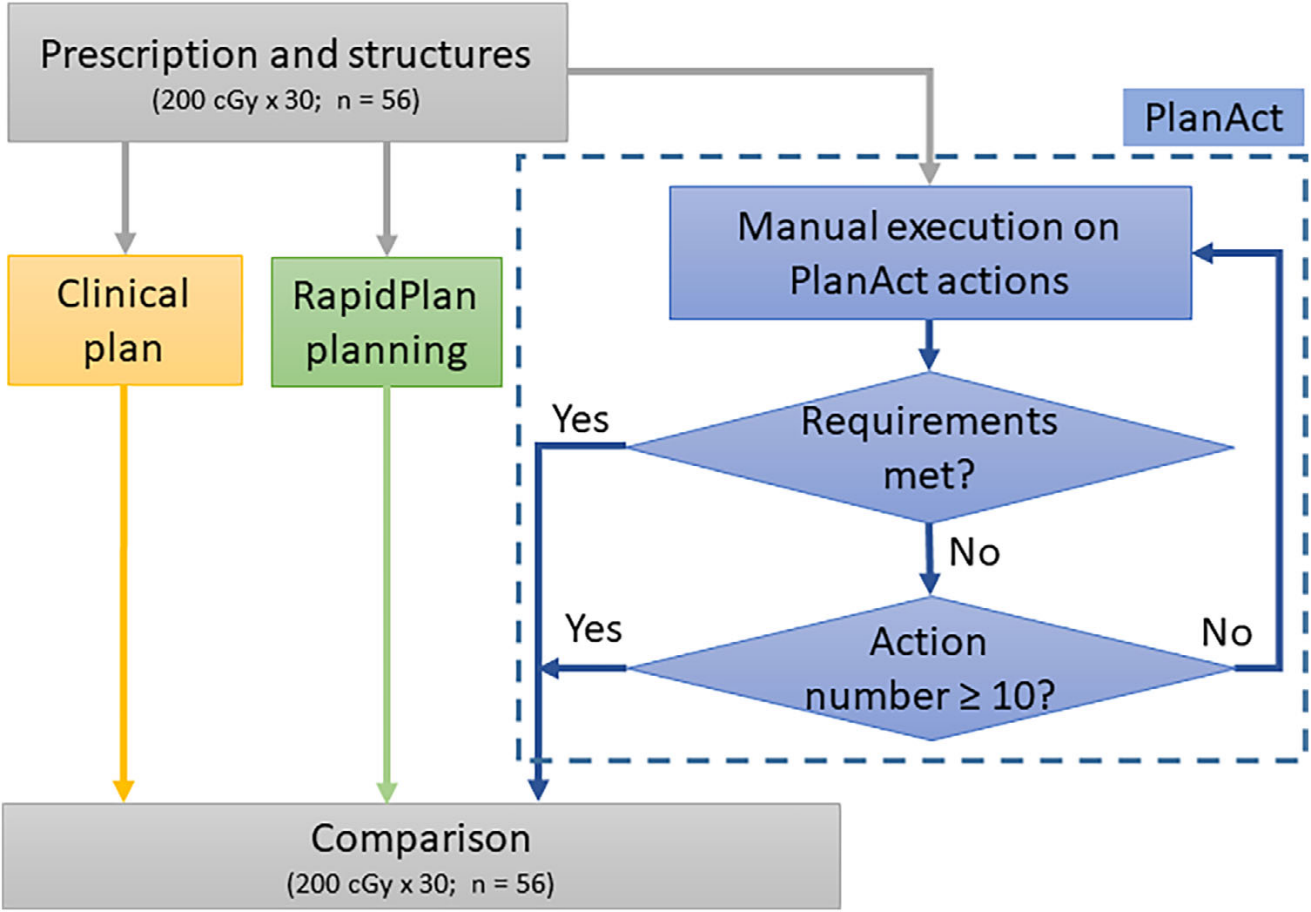
Workflow of the present study. In this study, “action number” refers to the sequential index of optimization actions applied to a treatment plan following its initial generation by knowledge‐based planning.

We retrieved 56 anonymized stage II or IIIA LA‐NSCLC cases previously treated between November 2022 and January 2024 at our institution. The mean PTV volume of the studied cases is 402.4 cc, with a standard deviation of 218.0 cc. The 56 cases included VMAT (volumetric‐modulated arc therapy) clinical plans approved for treatment. Experimental IMRT treatment plans were generated using nine static beams positioned at standardized consistent gantry angles at 160°, 120°, 80°, 40°, 0°, 320°, 280°, 240°, and 200°. The prescribed dose was the same across all plans within the cohort at 200 cGy × 30 fractions. The planning was conducted using a custom‐developed ESAPI‐based scripting module, named PlanAct, which was evaluated against both original clinical plans and KBP‐based plans generated by RapidPlan. The overall workflow is illustrated in Figure 1. We compared the dosimetric performance across three planning strategies—clinical plans, RapidPlan‐generated plans, and PlanAct‐optimized plans. All plans were normalized to ensure 95% PTV coverage at 100% of the prescription dose.

Plan generation involved manual iterative script executions (actions) with up to 10 cycles (action number ≤ 10) performed when clinical dosimetric objectives (as listed in Table 2) were not initially met. A human planner selected an action and decided corresponding parameters (OAR_structure, target_dose_level, constraint_weighting, etc.) in each cycle. During initialization (by the Initializer), the PTV was cropped by subtracting its overlap with small OARs (spinal cord, esophagus, larynx). For large OARs (lungs, heart), we did not crop the PTV; instead, we tightened OAR constraints (e.g., Dmean) stepwise while maintaining the PTV dose coverage. The PlanAct scripting module was implemented using ESAPI version 15.6 (Varian Medical Systems, Palo Alto, CA) and compiled with the .NET framework version 4.7.2. All optimizations were run on a dual‐socket Intel Xeon Silver 4110 (2.10 GHz) workstation, 32 GB RAM, Windows 10 Enterprise 2016, Eclipse 15.6 (Photon Optimizer 15.6.03), with final dose computed using AAA 15.6.03.

**TABLE 2:** Clinical evaluation criteria used in this study.

Structure	DVH objective	Description	Goal
PTV	D_95_	Relative dose covering 95% of volume	≥ 100% of prescription dose
PTV	D_0.03cc_	Relative dose to 0.03 cc volume	≤ 110% of prescription dose
Plan (all structures)	D_0.03cc_	Relative dose to 0.03 cc volume	≤ 110% of prescription dose
Lungs	V_20_	Percentage of volume receiving ≥ 20 Gy	≤ 37% of structure volume
Lungs	D_mean_	Mean dose to structure	≤ 2000 cGy
Heart	D_50_	Dose covering 50% of volume	≤ 3000 cGy
Spinal cord	D_0.03cc_	Dose to 0.03 cc volume	≤ 4500 cGy
Esophagus	D_mean_	Mean dose to structure	≤ 3400 cGy
Esophagus	D_0.03cc_	Dose to 0.03 cc volume	≤ 6600 cGy
Larynx	D_3cc_	Dose to 3 cc volume	≤ 4000 cGy
Larynx	D_0.03cc_	Dose to 0.03 cc volume	≤ 4600 cGy

### 2.3 Plan quality metrics

To evaluate plan quality, we used our institutional plan quality metrics, which were established based on published literature.^13^, ^14^, ^15^, ^16^ These metrics included PTV coverage, maximum dose (defined as D_0.03cc_ for OARs), and OAR constraints for structures such as the esophagus, lungs, heart, larynx, and spinal cord (Table 2). Dose‐volume histogram (DVH) objectives served as the primary tool for assessing treatment plan compliance with these clinical goals.

### 2.4 Statistical analysis

All statistical analyses were performed using Stata Version 17.0 Basic Edition (StataCorp LLC, TX, USA). The paired Wilcoxon signed‐rank test was used to evaluate a null hypothesis that the median difference between paired samples was zero. A *p*‐value < 0.05 was considered statistically significant to reject the null hypothesis.

## 3 RESULTS

**FIGURE 2:**
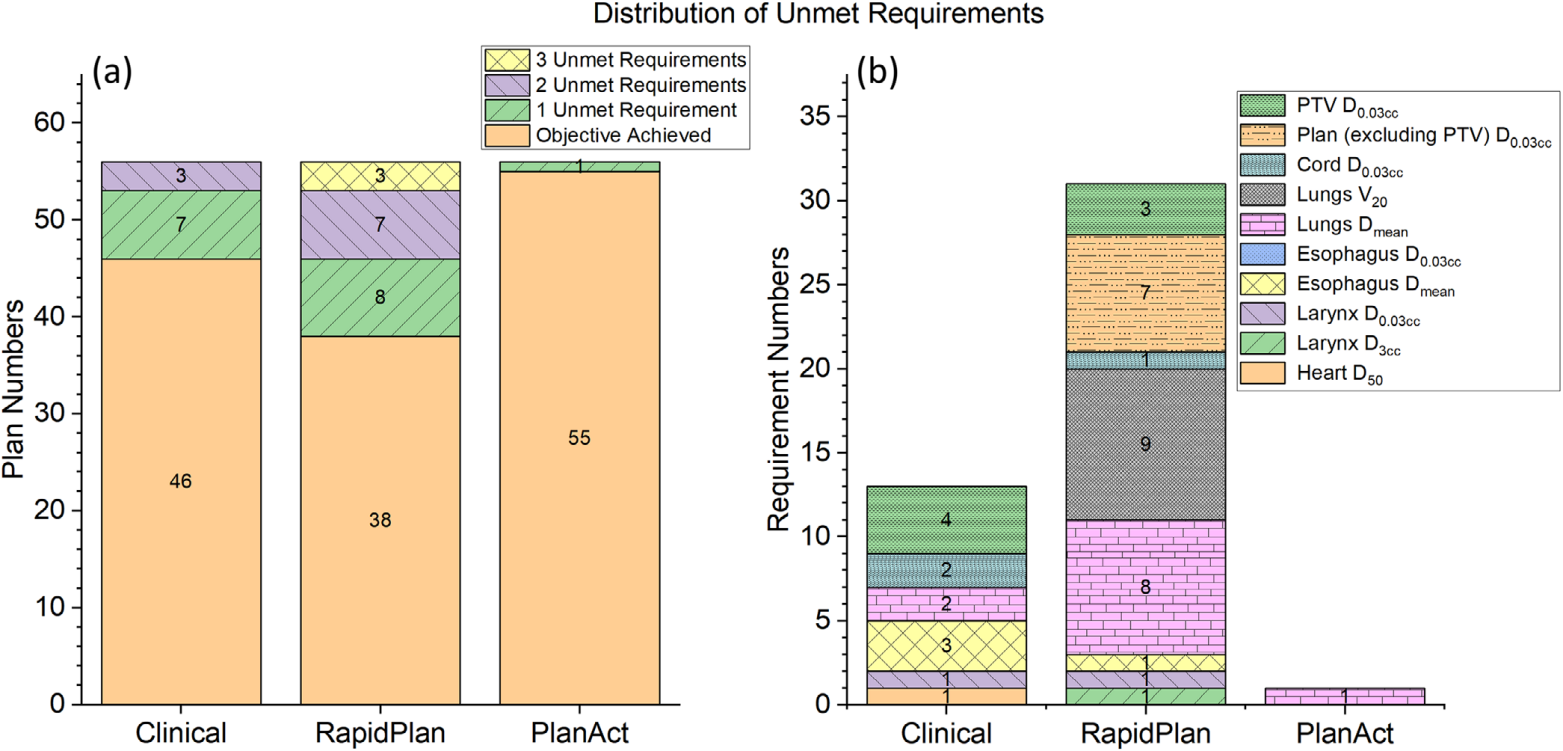
Comparison of unmet dosimetric requirements among clinical, RapidPlan‐generated, and PlanAct‐optimized IMRT plans across 56 LA‐NSCLC cases, including (a) the number of plans with 0, 1, 2, or 3 unmet clinical DVH requirements, and (b) the distribution of specific unmet DVH constraints across all cases. Each color in panel (b) corresponds to a different dosimetric metric.

Figure 2 shows that the number of unmet dosimetric requirements per case was lower in PlanAct‐optimized plans compared to both RapidPlan‐generated and clinical plans. While 18 RapidPlan‐generated and 10 clinical plans had at least one unmet criterion, only one PlanAct plan failed (in lungs D_mean_) to fully achieve the DVH objectives described in Table 2, indicating superior overall compliance with clinical constraints. In the other 55 cases, PlanAct achieved full compliance, demonstrating superior overall adherence to clinical requirements. The clinical plans exhibited suboptimal dosimetric outcomes primarily in terms of PTV D_0.03cc_ and esophagus dose constraints, whereas the RapidPlan‐generated plans underperformed predominantly concerning lung doses and Plan D_0.03cc_, particularly in regions external to the PTV.

**FIGURE 3:**
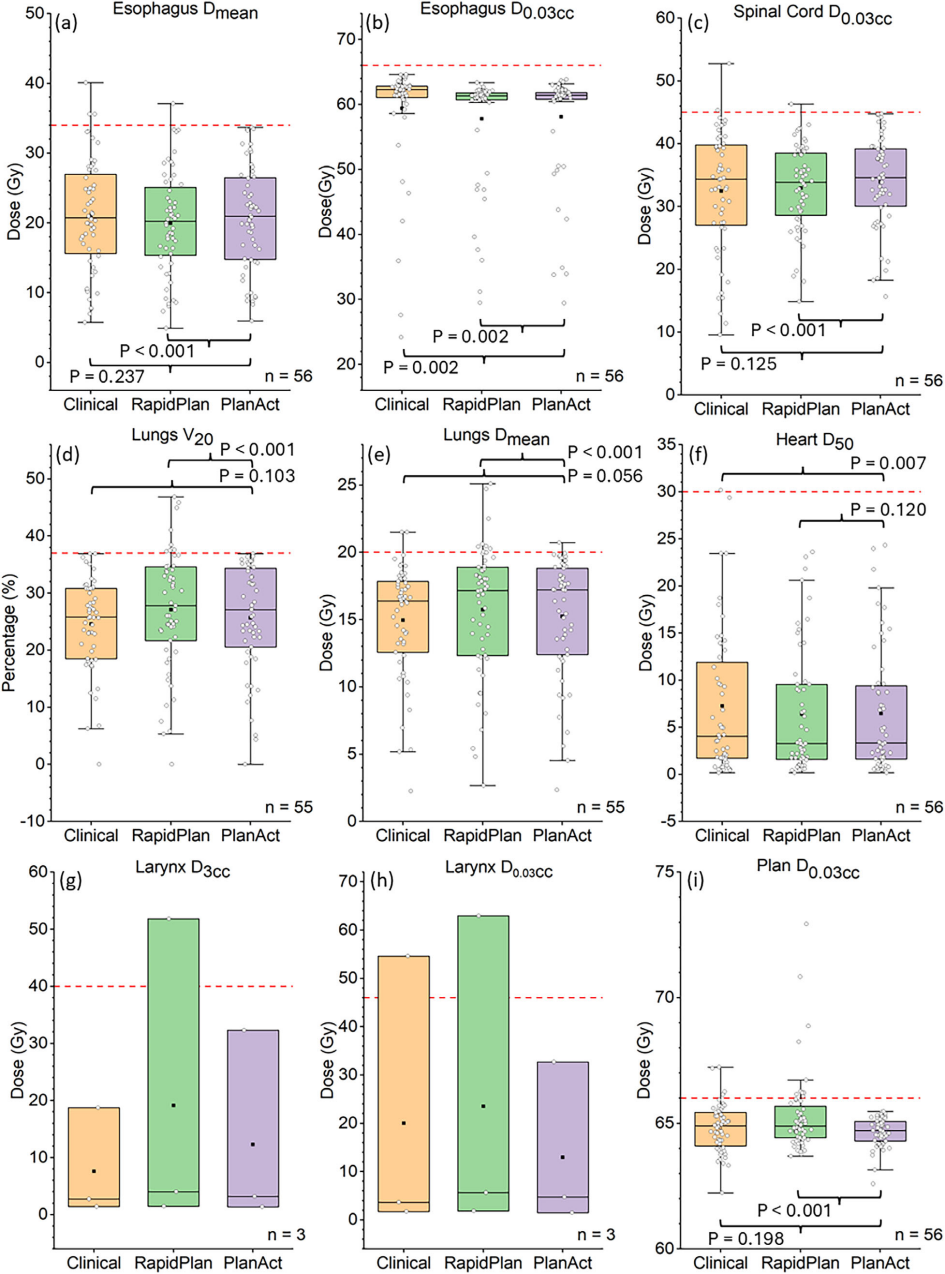
Comparison of dosimetric parameters between RapidPlan‐generated and PlanAct‐optimized IMRT plans across 56 LA‐NSCLC cases, including (a) esophagus Dmean, (b) esophagus D_0.03cc_, (c) spinal cord D_0.03cc_, (d) lungs V_20_, (e) lungs D_mean_, (f) heart D_50_, (g) larynx D_3cc_, (h) larynx D_0.03cc_, (j) PTV D_95_, (k) PTV D_0.03cc_, and (i) Plan (body) D_0.03cc_. The horizontal dashed lines indicate the DVH objectives in each metric. P‐values in subfigures represent results of the paired Wilcoxon signed‐rank test.

Compared with the original clinical plans, PlanAct reliably eliminated overdose regions and maintained either statistically similar (*p* > 0.05) or significantly lower (*p* < 0.05) median dose exposure to most OARs, including the esophagus, lungs, spinal cord, heart, and body, as shown in Figure 3. In direct comparisons between RapidPlan‐generated and PlanAct‐optimized plans, PlanAct was able to reduce mean doses to the lungs and maximum doses to the body, with the cost of higher esophagus D_mean_ and spinal cord D_0.03cc_. Although statistical analysis was not feasible for the larynx due to limited data, all three cases with contoured larynx structures in the PlanAct group achieved both D_3cc_ and D_0.03cc_ objectives as shown in Figure 3g,h. In contrast, one plan each from the clinical and RapidPlan groups failed to meet at least one larynx dose constraint.

**FIGURE 4:**
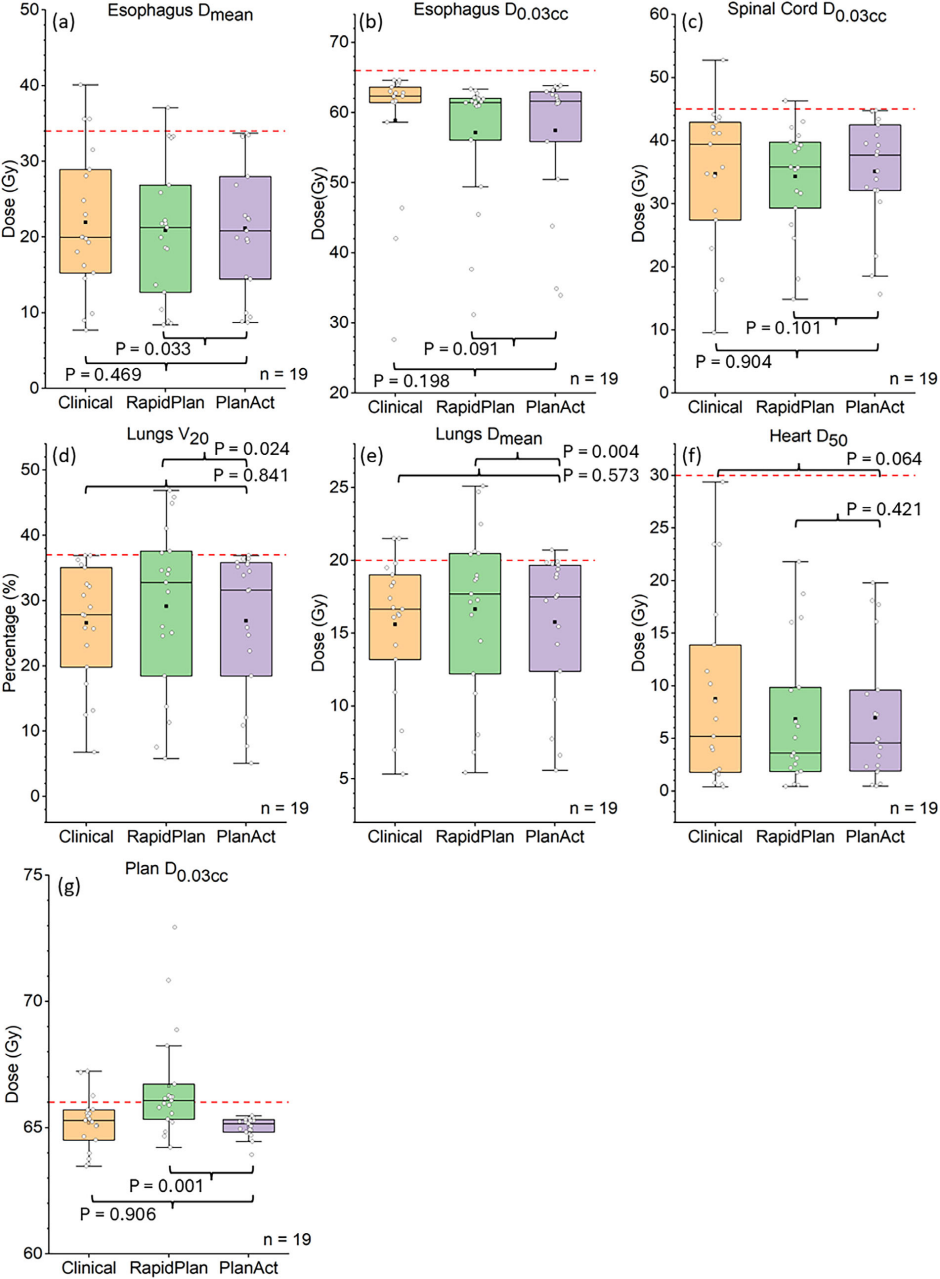
Comparison of dosimetric parameters among RapidPlan‐generated and PlanAct‐optimized IMRT plans across 19 LA‐NSCLC cases in which PlanAct was iterated at least once, including (a) esophagus D_mean_, (b) esophagus D_0.03cc_, (c) spinal cord D_0.03cc_, (d) lungs V_20_, (e) lungs D_mean_, (f) heart D_50_, and (g) Plan (body) D_0.03cc_. The horizontal dashed lines indicate the DVH objectives in each metric. *p*‐values in subfigures represent results of the paired Wilcoxon signed‐rank tests. Only one (out of 19) case had a contoured larynx structure, the dose results of which are shown in Figure 3 g,h.

For the 19 cases that PlanAct was iterated at least once after plan initialization, there were no statistically significant differences in all metrics between the original clinical plans and the PlanAct‐optimized plans. As shown in Figure 4, compared with the RapidPlan‐generated plans, PlanAct reliably eliminated overdose to all structures and significantly reduced lungs D_mean_ and the body D_0.03cc_ (*p* < 0.05), confirming that the findings from Figure 3 hold true in challenging cases.

**FIGURE 5:**
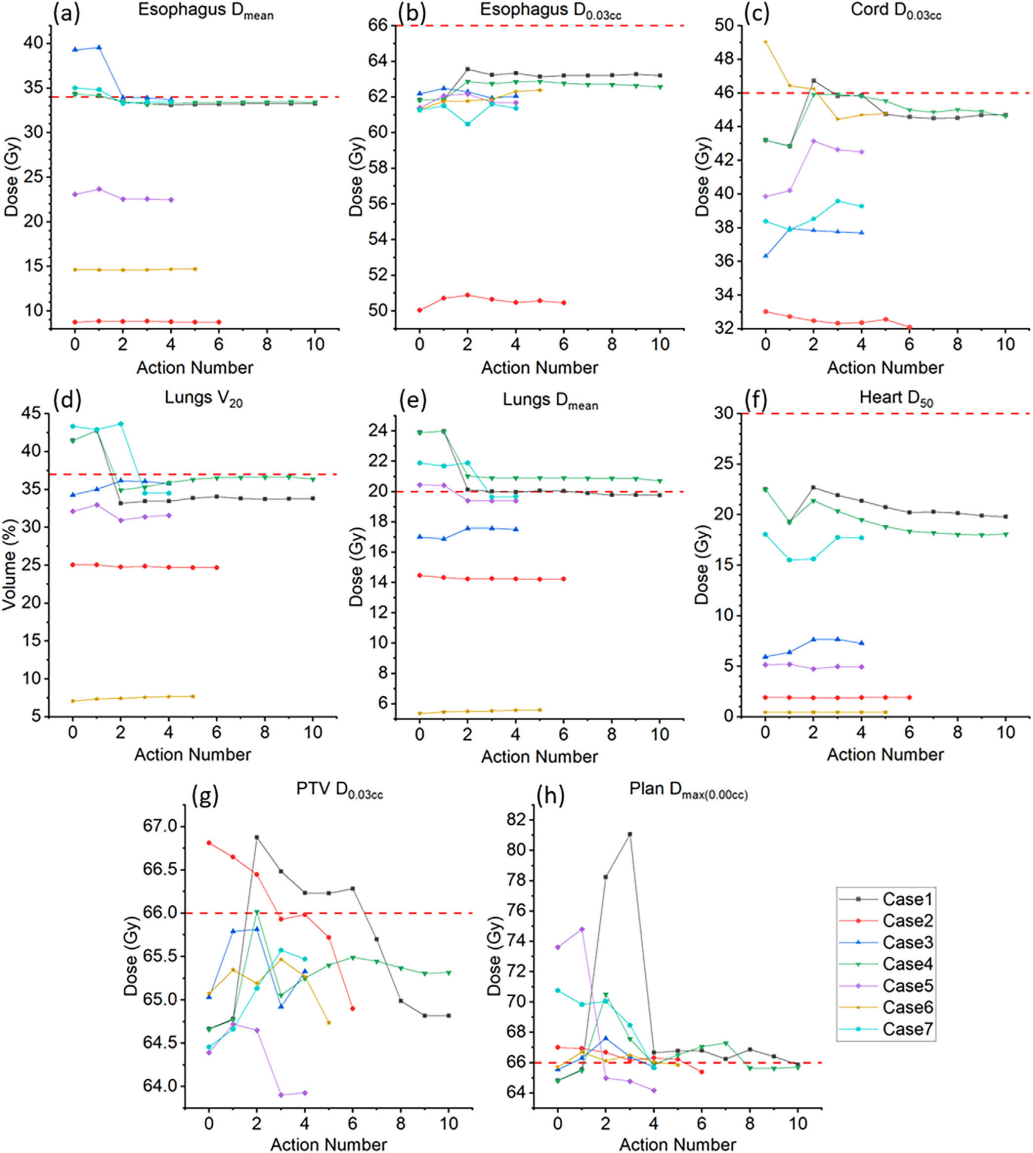
Dose variation of dosimetric metrics across sequential optimization actions for individual cases. Each line represents a distinct case, with the horizontal axis indicating action number and the vertical axis indicating absolute dose (Gy) or volume fraction (%) of a structure.

Figure 5 shows sequential optimization actions in the most challenging cases in which PlanAct actions were executed at least four times (excluding Initializer). Action number 0 corresponds to the pre‐optimization plan (i.e., immediately after the Initializer). Subsequent numbers (1, 2, 3, etc.) represent sequential optimization steps (after initialization) applied through PlanAct's modular scripts to achieve dosimetric objectives. Overall, the action sequence effectively balanced OAR sparing with target dose conformity, similarly to sequential progress human planners make in handling complex cases in manual optimization. In the majority of cases, a total of four actions (each action took approximately three minutes) were sufficient to optimize a pre‐satisfactory plan generated by KBP.

Despite one PlanAct‐optimized plan not fully meeting lung protection objectives, a closer analysis reveals that these limitations were attributable to a large PTV volume (770.72 cc). As shown in Table 3, the PlanAct‐optimized plan missed only one constraint (lung D_mean_ = 21.1%), compared to three unmet requirements in the RapidPlan‐generated plan (including a much higher D_mean_ of 25.1%) and two in the clinical plan. The PlanAct‐optimized plan also achieved the lowest lungs V_20_ and heart D_50_ (17.6%) and the most favorable hotspot control (PTV D_0.03cc_ = 108.0% = 64.8 Gy; body D_0.03cc_ = 108.7% = 65.2 Gy).

**TABLE 3:** Dosimetric comparison for the challenging case where the PlanAct‐optimized plans have an unmet DVH objective.

Structure	Unmet Requirements	Esophagus		Spinal Cord	Lungs		Heart	PTV		Body
	Number	D_mean_ (Gy)	D_0.03cc_ (Gy)	D_0.03_ (Gy)	D_mean_ (Gy)	V_20_ (%)	D_50_ (%)	D_95_ (%)	D_0.03cc_ (%)	D_0.03cc_ (Gy)
Clinical	2	**35.6**	64.6	41.2	**21.5**	36.9	23.5	100.0	109.4	65.7
RapidPlan	3	33.3	62.0	43.0	**25.1**	**45.9**	18.7	100.0	108.4	**68.9**
PlanAct	1	33.4	62.1	42.7	**21.1**	36.0	17.6	100.0	108.0	65.2

*Note*: The dosimetrically favorable values are underlined, and the values exceeding the DVH objectives are in **bold**.

**FIGURE 6:**
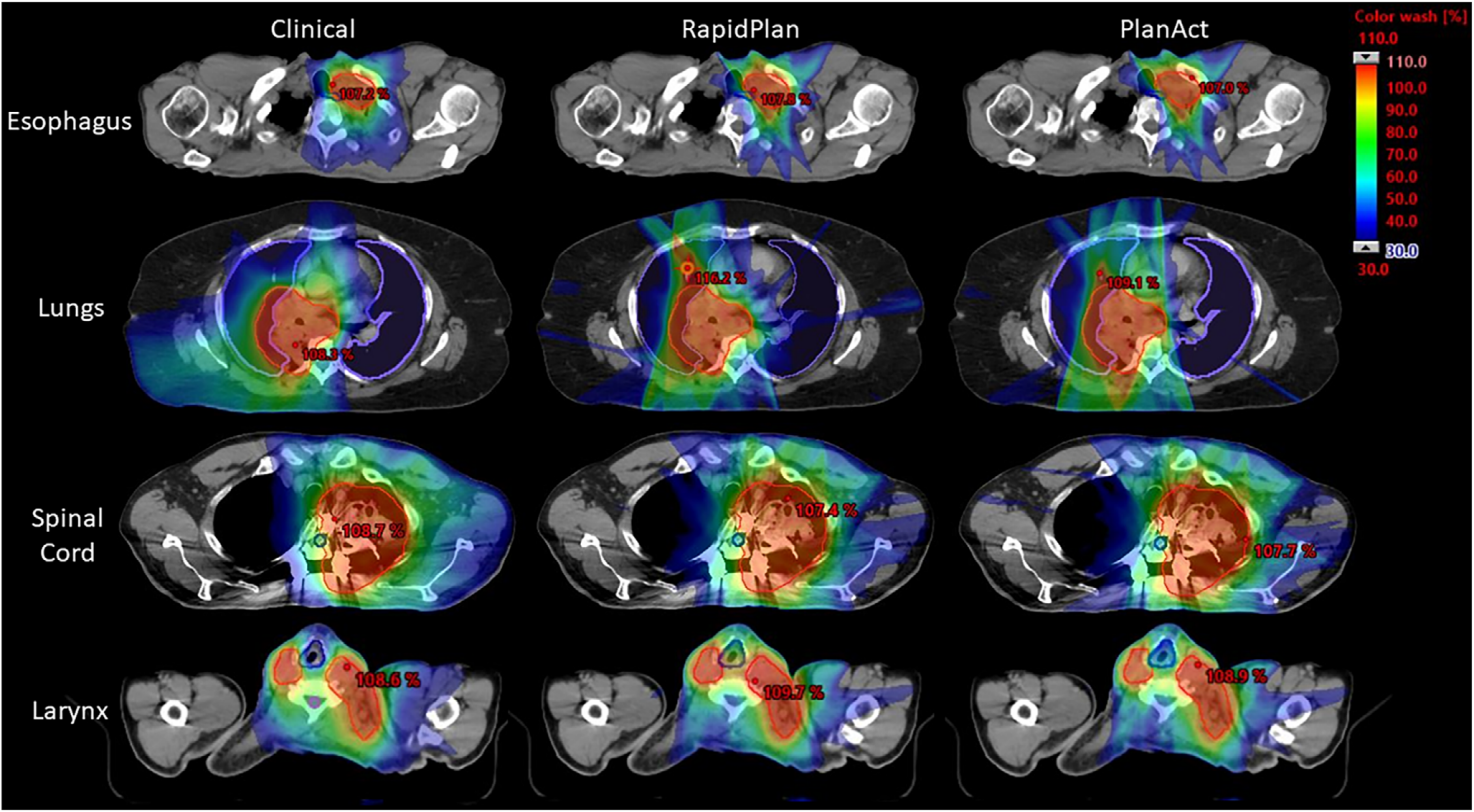
Transversal slice comparison of dose distributions in typical cases. Each row shows the same slice of the maximum dose to the indicated organs‐at‐risk. The original clinical plans were VMAT plans, while the RapidPlan‐generated and PlanAct‐optimized plans were IMRT plans. Red outlined contour: PTV. Blue/purple outlined contour: corresponding organs‐at‐risk.

Figure 6 presents a transversal slice in the treatment planning system from four cases in which the original clinical plans did not fully meet the dosimetric objectives (corresponding DVH comparisons are shown in Figure S1). The unmet objectives are esophagus D_0.03cc_, lungs D_0.03cc_ and D_mean_, spinal cord D_0.03cc_, and larynx D_3cc_. The RapidPlan‐generated plans were not able to achieve the unmet objectives. In comparison, the PlanAct‐optimized plan (the third column) reduced the extent of the hotspot, spreading doses to the adjacent volume around the hotspots.

## 4 DISCUSSION

This study proposed and validated an ESAPI‐based scripting module PlanAct, which embeds clinical strategies to optimize automated planning (RapidPlan) in LA‐NSCLC. Applied to 56 retrospective cases, PlanAct‐optimized (IMRT) plans demonstrated superior dosimetric performance and higher adherence to institutional dose constraints compared to both RapidPlan and clinical benchmarks. Only one PlanAct plan failed to meet one DVH objective (lungs D_mean_, due to a large PTV volume), whereas 18 RapidPlan (IMRT) and 10 clinical (VMAT) plans exhibited at least one violation. In the remaining 55 cases, PlanAct achieved complete compliance, underscoring its reliability in producing clinically acceptable plans across diverse anatomical presentations.

Although the VMAT and IMRT techniques are not identical, many studies have pointed out that both techniques offer similar dosimetric outcomes in LA‐NSCLC,^17^, ^18^, ^19^ supporting dosimetric comparability for LA‐NSCLC in our present study. Notably, PlanAct excelled in sparing healthy lung tissue, which is a persistent challenge in LA‐NSCLC due to large PTV overlap volume. Statistically significant reductions in lung V_20_ and D_mean_ were observed (*p* < 0.001), with PlanAct outperforming RapidPlan across all cases. Lung V_5_ was not evaluated in this study as it is not part of our clinical dose constraints. The single PlanAct plan that exceeded the lung D_mean_ threshold was attributed to anatomical constraints, not algorithmic limitations, and still achieved superior overall dose distributions to the lungs and heart, compared with the clinical plan and RapidPlan‐generated plan. Across other key metrics, including esophagus D_mean_ and D_0.03cc_, spinal cord D_0.03cc_, and larynx D_3cc_ and D_0.03cc_, PlanAct matched or exceeded the performance of comparator methods. Although there might be trade‐offs such as increased plan complexity (e.g., higher monitor units and modulation), PlanAct's ability to consistently minimize high‐dose hotspots and reduce the number of unmet planning criteria affirms its potential in clinical utility, particularly in high‐throughput settings or institutions with variable planning expertise.

RapidPlan is a KBP system that applies a static, one‐step optimization using DVH predictions learned from a library of prior clinical plans. Once a RapidPlan model is applied, its predicted objectives are used to generate the plan in a single pass, without contextual adaptation during the planning process. In contrast, human planners typically approach treatment planning as a dynamic, feedback‐driven process. They iteratively evaluate the plan's dosimetric results, identify underperformance or constraint violations, and selectively adjust objectives or structure priorities to guide the optimizer. PlanAct is designed to replicate this human‐in‐the‐loop strategy by decomposing the planning process into sequential modules, each targeting a specific clinical objective (e.g., undercoverage, high OAR dose, hotspots). After each step, the plan is re‐evaluated, and subsequent actions are informed by the updated dosimetric context. This iterative workflow allows PlanAct to flexibly address case‐specific challenges and adjust to anatomical variability, enabling improvements on RapidPlan‐generated plans.

The proposed method addresses two major barriers preventing clinical adoptions of RapidPlan models. First, RapidPlan models require extensive tuning in order to generate clinical plans consistently. The model‐tuning process is highly time‐consuming and labor‐intensive, as it requires multiple iterations of testing constraint settings on a cohort of patients.^20^, ^21^ Such efforts prevent many institutions from fully implementing RapidPlan in clinical environments. The proposed PlanAct showcases an alternative approach, where PlanAct allows the model‐generated plans to conform to institution‐specific requirements, reducing the reliance on institution‐specific models and potentially enabling direct adoption of published models by other institutions. Second, plans generated by RapidPlan models, especially the ones that prioritize OAR sparing tend to require one or more iterations of planning interactions to reduce max dose and remove hotspots outside the PTV. The proposed PlanAct automates the process reliably, thereby further expanding the benefits of adopting RapidPlan models in the clinic.

Recent advances in automated and knowledge‐based planning have significantly enhanced the efficiency, consistency, and quality of radiotherapy treatment planning.^22^, ^23^ The modular architecture of PlanAct, where individual scripts systematically address specific optimization tasks such as constraint application, substructure delineation, and hotspot management, facilitates interpretability and flexibility in clinical use. This structured approach closely mirrors expert planners' reasoning, enabling standardized treatment quality across diverse patient anatomies and varying clinical protocols.

PlanAct's design could further support seamless integration into future automated planning platforms driven by artificial intelligence (AI).^24^ PlanAct is designed for automation. For this study, we kept a human‐in‐the‐loop to review unmet constraints and select the next action/parameters in order to validate the capability of the PlanAct platform for generating clinically acceptable treatment plans, which is the first step to validate the platform before automation. The same logic is batch‐automatable, enabling a future AI application to choose actions/parameters automatically under the same dosimetric constraints. By systematically addressing discrete planning tasks and providing immediate dose‐volume feedback, PlanAct can serve as an effective framework for intelligent planning agents. For example, language model‐driven systems like GPT‐Plan and GPT‐RadPlan have been demonstrated their capability to emulate the reasoning of expert planners;^25^, ^26^ however, due to the stochastic nature of large language models, they could not avoid occasional hallucinations which can lead to suboptimal or even unsafe treatment plans. As PlanAct embeds clinical optimization strategies, it could potentially be driven by AI agents, with mitigated risks associated with AI's hallucinations, to achieve full autonomous planning. Future developments may focus on combining PlanAct with advanced AI techniques to further reduce planning time and enhance consistency. Additionally, deploying such standardized tools in multi‐institutional settings could enable robust benchmarking and continuous improvement of planning practices, supporting broader and high‐quality care across diverse clinical environments.

## 5 CONCLUSION

In this study, we developed and validated PlanAct, an ESAPI‐based scripting module designed to optimize automated IMRT planning in NSCLC. PlanAct consistently delivered superior or comparable dosimetric quality relative to RapidPlan‐generated and clinical plans, effectively reducing unmet dose‐volume objectives and enhancing organ‐at‐risk sparing. The modular design of PlanAct allows systematic refinement of treatment plans, thus facilitating standardization and increased efficiency in human‐driven planning workflows.

## AUTHOR CONTRIBUTIONS

**Hao Guo, Yang Lei, Kenneth Rosenzweig, Robert Samstein, Ming Chao, Tian Liu, Junyi Xia, and Jiahan Zhang**: Conceived and designed the study. **Hao Guo and Jiahan Zhang**: Acquired the data. **Hao Guo, Junyi Xia, and Jiahan Zhang**: Performed data analysis and interpretation, and drafted the manuscript, contributing significant intellectual content. **Ming Chao, Tian Liu, Junyi Xia, and Jiahan Zhang**: Supervised the work, providing oversight and guidance to trainees. **Hao Guo, Tenzin Kunkyab, Kenneth Rosenzweig, Tian Liu, Junyi Xia, and Jiahan Zhang**: Critically revised the manuscript. All authors have reviewed and approved the final version for publication.

## ETHIC STATEMENT

This study was approved by an Institutional Review Board of the Mount Sinai School of Medicine (STUDY‐23‐01493‐MODCR001).

## CONFLICT OF INTEREST STATEMENT

The authors declare no conflicts of interest.

## GENERATIVE AI AND LARGE LANGUAGE MODELS

During the preparation of this work the authors used Chat GPT‐4o (OpenAI Inc., San Francisco, CA) in order to improve the language quality. After using this tool, the authors reviewed and edited the content as needed and take full responsibility for the content of the publication.

## Supporting information



Supporting Information

Supporting Information

## Data Availability

The data that support the findings of this study are available on request from the corresponding author. The data are not publicly available due to privacy or ethical restrictions.

## References

[1] Grégoire V , Langendijk JA , Nuyts S . Advances in radiotherapy for head and neck cancer. JCO. 2015;33(29):3277‐3284. doi:10.1200/JCO.2015.61.2994 10.1200/JCO.2015.61.2994 26351354

[2] Fornacon‐Wood I , Chan C , Bayman N , et al. Impact of introducing intensity modulated radiotherapy on curative intent radiotherapy and survival for lung cancer. Front Oncol. 2022;12:835844. doi:10.3389/fonc.2022.835844 35712515 10.3389/fonc.2022.835844 PMC9197586

[3] Chan C , Lang S , Rowbottom C , Guckenberger M , Faivre‐Finn C . Intensity‐modulated radiotherapy for lung cancer: current status and future developments. J Thorac Oncol. 2014;9(11):1598‐1608. doi:10.1097/JTO.0000000000000346 25436795 10.1097/JTO.0000000000000346

[4] Zarepisheh M , Hong L , Zhou Y , et al. Automated and clinically optimal treatment planning for cancer radiotherapy. INFORMS J Appl Anal. 2022;52(1):69‐89. doi:10.1287/inte.2021.1095 35847768 10.1287/inte.2021.1095 PMC9284667

[5] Berry SL , Boczkowski A , Ma R , Mechalakos J , Hunt M . Interobserver variability in radiotherapy plan output: results of a single‐institution study. Pract Radiat Oncol. 2016;6(6):442‐449. doi:10.1016/j.prro.2016.04.005 27374191 10.1016/j.prro.2016.04.005 PMC5099085

[6] Nelms BE , Robinson G , Markham J , et al. Variation in external beam treatment plan quality: an inter‐institutional study of planners and planning systems. Pract Radiat Oncol. 2012;2(4):296‐305. doi:10.1016/j.prro.2011.11.012 24674168 10.1016/j.prro.2011.11.012

[7] Hussein M , Heijmen BJM , Verellen D , Nisbet A . Automation in intensity modulated radiotherapy treatment planning—a review of recent innovations. Br J Radiol. 2018;91(1092):20180270. doi:10.1259/bjr.20180270 30074813 10.1259/bjr.20180270 PMC6319857

[8] Cao W , Gronberg M , Olanrewaju A , et al. Knowledge‐based planning for the radiation therapy treatment plan quality assurance for patients with head and neck cancer. J Appl Clin Med Phys. 2022;23(6):e13614. doi:10.1002/acm2.13614 35488508 10.1002/acm2.13614 PMC9195018

[9] Fogliata A , Cozzi L , Reggiori G , et al. RapidPlan knowledge based planning: iterative learning process and model ability to steer planning strategies. Radiation Oncology. 2019;14(1):187. doi:10.1186/s13014‐019‐1403‐0 31666094 10.1186/s13014-019-1403-0 PMC6822368

[10] Kim H , Kwak J , Jeong C , Cho B . Institutional applications of eclipse scripting programming interface to clinical workflows in radiation oncology. Prog Med Phys. 2017;28(3):122‐128. doi:10.14316/pmp.2017.28.3.122

[11] Cardenas CE , Cardan RA , Harms J , Simiele E , Popple RA . Knowledge‐based planning, multicriteria optimization, and plan scorecards: a winning combination. Radiotherapy and Oncology. 2025:202. doi:10.1016/j.radonc.2024.110598 10.1016/j.radonc.2024.110598 PMC11663123 39490417

[12] Kunkyab T , Lei Y , Guo H , et al. Knowledge‐based trade‐off prediction for NSCLC treatment planning using multi‐output regression. Medical Physics. 2025;52(9):e18068. doi:10.1002/mp.18068 40849864 10.1002/mp.18068

[13] Bisello S , Cilla S , Benini A , et al. Dose–volume constraints fOr oRganS at risk in radiotherapy (CORSAIR): an “All‐in‐One” multicenter–multidisciplinary practical summary. Curr Oncol. 2022;29(10):7021‐7050. doi:10.3390/curroncol29100552 36290829 10.3390/curroncol29100552 PMC9600677

[14] Marks LB , Bentzen SM , Deasy JO , et al. Radiation dose volume effects in the lung. Int J Radiat Oncol Biol Phys. 2010;76:S70‐S76. doi:10.1016/j.ijrobp.2009.06.091 20171521 10.1016/j.ijrobp.2009.06.091 PMC3576042

[15] Levin N , Killingberg KT , Halvorsen TO , Danielsen S , Grønberg BH . Evaluation of radiation therapy treatment plans in a randomized phase 2 trial comparing 2 schedules of twice‐daily thoracic radiation therapy in limited stage small cell lung cancer. Int J Radiat Oncol Biol Phys. 2024;120(2):332‐342. doi:10.1016/j.ijrobp.2024.03.045 38583494 10.1016/j.ijrobp.2024.03.045

[16] Fleming C , Cagney DN , O'Keeffe S , Brennan SM , Armstrong JG , McClean B . Normal tissue considerations and dose–volume constraints in the moderately hypofractionated treatment of non‐small cell lung cancer. Radiother Oncol. 2016;119(3):423‐431. doi:10.1016/j.radonc.2016.03.013 27084120 10.1016/j.radonc.2016.03.013

[17] Ata RSES , Gad OAEF , Sebaie MME , El‐Kady AM . Comparison between intensity‐modulated radiotherapy (IMRT) and volumetric modulated arc therapy (VMAT) in the treatment of locally advanced non‐small cell lung cancer. J Popl Ther Clin Pharmacol. 2025;32(1):520‐535. doi:10.53555/hr4af222

[18] Li C , Luo H , Song W , Hu Y , Li J , Cai Z . Dosimetric comparison of four radiotherapy techniques for stage III non‑small cell lung cancer. Oncol Lett. 2023;26(2):347. doi:10.3892/ol.2023.13933 37427336 10.3892/ol.2023.13933 PMC10326827

[19] Jiang X , Li T , Liu Y , et al. Planning analysis for locally advanced lung cancer: dosimetric and efficiency comparisons between intensity‐modulated radiotherapy (IMRT), single‐arc/partial‐arc volumetric modulated arc therapy (SA/PA‐VMAT). Radiat Oncol. 2011;6(1):140. doi:10.1186/1748‐717X‐6‐140 22014217 10.1186/1748-717X-6-140 PMC3207896

[20] Harms J , Pogue JA , Cardenas CE , Stanley DN , Cardan R , Popple R . Automated evaluation for rapid implementation of knowledge‐based radiotherapy planning models. J Appl Clin Med Phys. 2023;24(10):e14152. doi:10.1002/acm2.14152 37703545 10.1002/acm2.14152 PMC10562024

[21] Fogliata A , Cozzi L , Reggiori G , et al. RapidPlan knowledge based planning: iterative learning process and model ability to steer planning strategies. Radiother Oncol. 2019;14(1):187. doi:10.1186/s13014‐019‐1403‐0 10.1186/s13014-019-1403-0 PMC6822368 31666094

[22] Chung CV , Khan MS , Olanrewaju A , et al. Knowledge‐based planning for fully automated radiation therapy treatment planning of 10 different cancer sites. Radiother Oncol. 2025:202. doi:10.1016/j.radonc.2024.110609 10.1016/j.radonc.2024.110609 39486482

[23] Momin S , Fu Y , Lei Y , et al. Knowledge‐based radiation treatment planning: a data‐driven method survey. J Appl Clin Med Phys. 2021;22(8):16‐44. doi:10.1002/acm2.13337 10.1002/acm2.13337 PMC8364264 34231970

[24] Sheng Y , Zhang J , Ge Y , et al. Artificial intelligence applications in intensity modulated radiation treatment planning: an overview. Quant Imaging Med Surg. 2021;11(12):4859‐4880. doi:10.21037/qims‐21‐208 34888195 10.21037/qims-21-208 PMC8611458

[25] Wang Q , Wang Z , Li M , et al. A feasibility study of automating radiotherapy planning with large language model agents. Phys Med Biol. 2025;12. doi:10.1088/1361‐6560/adbff1 10.1088/1361-6560/adbff1 40073507

[26] Liu S , Pastor‐Serrano O , Chen Y , et al. Automated radiotherapy treatment planning guided by GPT‐4Vision. arXiv. 2024. doi:10.48550/arXiv.2406.15609 10.1088/1361-6560/adf02c 40664228

