# Supplementary material for: PlanAct: An eclipse scripting API‐based module embedding clinical optimization strategies for automated planning in locally advanced non‐small cell lung cancer
Source: J Appl Clin Med Phys. 2025 Oct 9;26(10):e70304. doi: 10.1002/acm2.70304 (PMC12509247; doi:10.1002/acm2.70304)
Supplement: Supplementary file 1 — Supporting Information [file ACM2-26-e70304-s002.docx]

Pseudo codes of PlanAct actions

1. **Initializer**

MAIN(…):

PARSE input arguments: patientID, courseID, planID, Plan type, OAR preference, preference level

IF required arguments missing:

EXIT with error

CREATE ESAPI application

CALL Execute(...) with parsed arguments

FUNCTION Execute(...):

VALIDATE plan_type and OAR_preference

LOAD patient, course, plan, and structure set

INITIALIZE a new external beam plan (prescription = 60 Gy / 30 fx)

ADD beams to plan based on plan_type:

- IMRT: 9 static beams

- full_arc: 3 full arcs

- else: copy arcs from original plan

FIND structures: PTV, OARs (esophagus, heart, lungs, spinal cord, etc.)

ADD Ring and InnerRing structures if missing

MATCH model structures to patient structures

CALCULATE DVH estimates using RapidPlan model

EXTRACT key metrics:

- cord D0.03cc, ptv D0.03cc

- lungs V20, Dmean; heart D50

- esophagus Dmean, D0.03cc; larynx Dmean, D3cc

IF any constraints violated:

CREATE PTV_Crop by subtracting OARs from PTV

UPDATE optimization target

RECALCULATE DVH estimates after cropping

ADD Normal Tissue Objective (NTO)

CONFIGURE optimization and dose calculation models

SET optimizer options

IF IMRT:

OPTIMIZE two-stage, CALCULATE dose, NORMALIZE

ELSE (VMAT):

ENABLE jaw tracking, OPTIMIZE two-stage, CALCULATE dose, NORMALIZE

LOG update message

SAVE plan

CLOSE patient

1. **NormalizeDmean**

MAIN(…):

PARSE input arguments: patientID, courseID, planID, PTV_structure, Dmean_objective

CREATE ESAPI application

CALL Execute(...) with parsed arguments

FUNCTION Execute(…):

OPEN plan and begin modifications

EXTRACT prescription dose

GET structure set

FIND PTV structure by name

IF PTV found:

NORMALIZE the plan based on the mean dose within the PTV

SAVE plan modifications

ELSE:

LOG and print PTV not found

LOG update message

SAVE modifications

CLOSE patient

1. **ReduceROIMean**

MAIN(…):

PARSE input arguments: patientID, courseID, planID, OAR_structure, target_dose_level, constraint_weighting, plan_type, continue_flag

CREATE ESAPI application

CALL Execute(...) with parsed arguments

FUNCTION Execute(...):

OPEN plan and begin modifications

FIND ROI structure by name

IF ROI found:

SET optimizer options

IF IMRT:

OPTIMIZE two-stage, CALCULATE dose, NORMALIZE

ELSE (VMAT):

ENABLE jaw tracking, OPTIMIZE two-stage, CALCULATE dose, NORMALIZE

ELSE:

LOG and print ROI not found

LOG update message

SAVE modifications

EXIT

CLOSE patient

1. **IncreaseCoverage**

MAIN(…):

PARSE input arguments: patientID, courseID, planID, PTV_structure, isodose_level, target_dose_level, constraint_weighting, plan_type, contourNum, continue_planning_flag

CREATE ESAPI application

CALL Execute(...) with parsed arguments

FUNCTION Execute(...):

OPEN plan and begin modifications

EXTRACT prescription dose

FIND PTV structure

CREATE or FIND isodose structure `z_boost<level>(<contourNum>)`

CONVERT dose to isodose shell using ConvertDoseLevelToStructure()

SUBTRACT inner core from outer shell (to get ring)

REMOVE previous optimization objective for that structure (if any)

ADD lower objective to new isodose structure `z_boost<level>(<contourNum>)` with target_dose_level

SET optimizer options

IF IMRT:

OPTIMIZE two-stage, CALCULATE dose, NORMALIZE

ELSE (VMAT):

ENABLE jaw tracking, OPTIMIZE two-stage, CALCULATE dose, NORMALIZE

LOG update message

SAVE modifications

CLOSE patient

1. **ReduceBodyDmax**

MAIN(…):

PARSE input arguments: patientID, courseID, planID, Body_structure, isodose_level, target_dose_level, constraint_weighting, plan_type, contourNum, continue_planning_flag

CREATE ESAPI application

CALL Execute(...) with parsed arguments

FUNCTION Execute(...):

OPEN plan and begin modifications

EXTRACT prescription dose

CREATE or FIND isodose structure “CoolBody<level>(<contourNum>)”

SET SegmentVolume from Body

CONVERT isodose level to structure

REMOVE previous constraints on CoolBody<level>(<contourNum>)

ADD UPPER point objective to CoolBody<level>(<contourNum>) with target_dose_level

SET optimizer options

IF IMRT:

OPTIMIZE two-stage, CALCULATE dose, NORMALIZE

ELSE (VMAT):

ENABLE jaw tracking, OPTIMIZE two-stage, CALCULATE dose, NORMALIZE

LOG update message

SAVE modifications

CLOSE patient

1. **ReducePTVDmax**

MAIN(…):

PARSE input arguments: patientID, courseID, planID, PTV_structure, isodose_level, target_dose_level, constraint_weighting, plan_type, contourNum, continue_planning_flag

CREATE ESAPI application

CALL Execute(...) with parsed arguments

FUNCTION Execute(...):

OPEN plan and begin modifications

EXTRACT prescription dose

CREATE or FIND isodose structure “CoolPTV<level>(<contourNum>)”

SET SegmentVolume from PTV

CONVERT isodose level to structure

REMOVE previous constraints on CoolPTV<level>(<contourNum>)

ADD UPPER point objective to CoolPTV<level>(<contourNum>) with target_dose_level

SET optimizer options

IF IMRT:

OPTIMIZE two-stage CALCULATE dose, NORMALIZE

ELSE (VMAT):

ENABLE jaw tracking, OPTIMIZE two-stage, CALCULATE dose, NORMALIZE

LOG update message

SAVE modifications

CLOSE patient

1. **ReduceDvolume**

MAIN(…):

PARSE input arguments: patientID, courseID, planID, PTV_structure, OAR_structure, dose_level, target_dose_level, constraint_weighting, plan_type, continue_planning_flag

CREATE ESAPI application

CALL Execute(...) with parsed arguments

FUNCTION Execute(...):

OPEN plan and begin modifications

GET prescription, structure set, log file

FIND PTV and OAR structures

GET current volume of OAR at dose_level

ADD optimization constraint:

- Upper constraint at dose_level with volume target_dose_level

SET optimizer options

IF IMRT:

OPTIMIZE two-stage, CALCULATE dose, NORMALIZE

ELSE (VMAT):

ENABLE jaw tracking, OPTIMIZE two-stage, CALCULATE dose, NORMALIZE

LOG update message

SAVE modifications

CLOSE patient

1. **OptimizePlan**

MAIN(…):

PARSE input arguments: patientID, courseID, planID, plan_type, continue_planning_flag

CREATE ESAPI application

CALL Execute(...) with parsed arguments

FUNCTION Execute(...):

IF patientID is invalid:

EXIT

OPEN plam and begin modifications

GET prescription, structure set

SET plan to Absolute dose mode

SET calculation options for optimizer (e.g., MRLevelAtRestart)

SET optimizer options

IF IMRT:

OPTIMIZE two-stage, CALCULATE dose, NORMALIZE

ELSE (VMAT):

ENABLE jaw tracking, OPTIMIZE two-stage, CALCULATE dose, NORMALIZE

CALCULATE dose

LOG update message

SAVE modifications

CLOSE patient

1. **NormalizePlan**

MAIN(…):

PARSE input arguments: patientID, courseID, planID, PTV_structure, PTV_coverage

CREATE ESAPI application

CALL Execute(...) with parsed args

FUNCTION Execute(...):

OPEN plan and begin modifications

NORMALIZE plan so PTV receives PTV_coverage% of prescription dose

LOG update message

SAVE modifications

CLOSE patient
