# Supplementary material for: PlanAct: An eclipse scripting API‐based module embedding clinical optimization strategies for automated planning in locally advanced non‐small cell lung cancer
Source: J Appl Clin Med Phys. 2025 Oct 9;26(10):e70304. doi: 10.1002/acm2.70304 (PMC12509247; doi:10.1002/acm2.70304)
Supplement: Supplementary file 2 — Supporting Information [file ACM2-26-e70304-s001.docx]

**Supplementary materials**

**
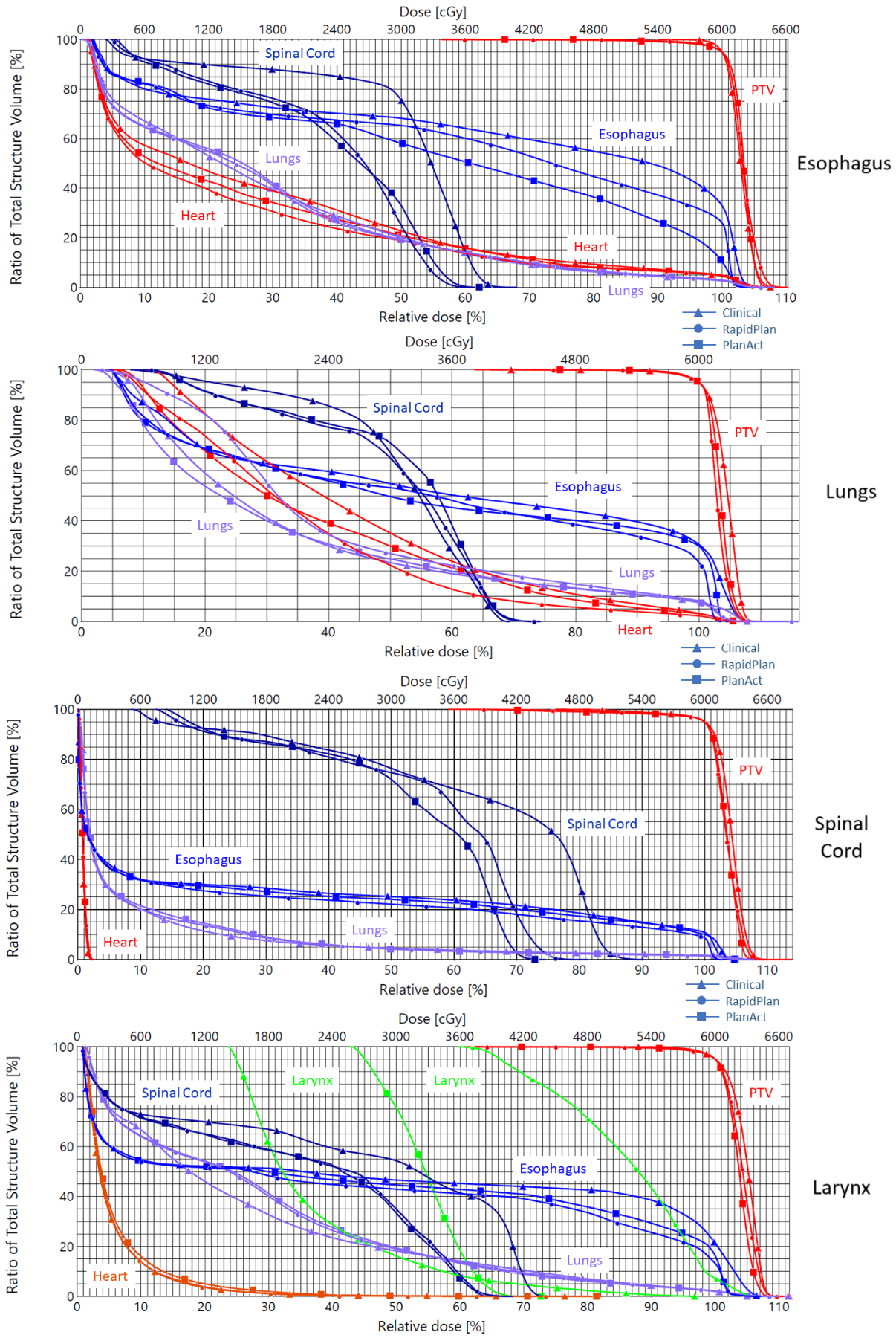
**

Figure S1. DVH comparison in typical cases shown in Figure 6. The original clinical plans (marked as triangle) were VMAT plans, while the RapidPlan-generated (marked as circle) and PlanAct-optimized (marked as square) plans were IMRT plans.
